# From the Classroom to Entrustment - The Development of Motivational Interviewing Skills as an Entrustable Professional Activity

**DOI:** 10.15694/mep.2019.000153.1

**Published:** 2019-07-17

**Authors:** Brett Engle, Kathryn Brogan-Hartlieb, Vivian T. Obeso, Maryse Pedoussaut, Michelle M. Hospital, Carla S. Lupi, Karin C. Esposito, David R. Brown

**Affiliations:** 1 Florida International University Herbert Wertheim College of Medicine; 2 Florida International University Community-Based Research Institute; 3 Kaiser Permanente School of Medicine

**Keywords:** Motivational Interviewing, Entrustable Professional Activity, Competency Based Medical Education, Patient Centered Behavioral Counseling, Communication Skills

## Abstract

This article was migrated. The article was marked as recommended.

Introduction

The move towards value-based care and population health has highlighted the prominent role of social and behavioral factors in determining health outcomes. Patient-centered behavioral guidance to improve patient self-management is recognized as an evidence-based intervention for a variety of chronic conditions but has yet to be adopted as a core competency or core entrustable professional activity (EPA). Motivational Interviewing (MI) is an evidence-based behavioral intervention involving an integrated set of competencies, featuring reflective listening, affirmation, evocation, and collaborative planning. An MI encounter is an observable, discrete task that can be framed as an EPA. Successful implementation of EPAs in the workplace requires institutional engagement, a thoughtful curricular approach, faculty development, and feasible, valid workplace-based assessment (WBA).

Methods

We implemented competency-based MI training and assessed competency outcomes for students and faculty. After joining the Association of American Medical Colleges Core EPA Pilot, we applied an iterative group process to develop an EPA and workplace-based assessment based on established MI competencies.

Results

Drawing upon nine years of developing MI curriculum, we present competency data for a student training study and a faculty coaching study, describe how we transitioned training from the classroom to the clinical setting employing an EPA framework, and present a one-page schematic and related WBA for an EPA based on MI.

Conclusion

We propose that MI is a core EPA for future physicians practicing value-based care, and offer a roadmap for curriculum implementation.

## Introduction

The move toward value-based care has highlighted social and behavioral determinants as principal drivers of health, making apparent that achieving greater value out of the health system requires new approaches built on social and behavioral foundations ( Irby, Cooke and O’Brien, 2010; Panel, 2011; Joynt *et al.*, 2017). Neither the most widely accepted competency frameworks nor the Association of American Medical Colleges (AAMC) Core Entrustable Professional Activities (EPAs) for Entering Residency effectively address behavior change counseling ( Englander *et al.*, 2013; Englander *et al.*, 2016). Since inception of the Herbert Wertheim College of Medicine (HWCOM) in 2009 ( Rock *et al.*, 2009) we have developed and implemented ( Brogan Hartlieb *et al.*, 2016) clinical skills training in MI to prepare our medical students for behavior change conversations during a required series of household visits ( Greer *et al.*, 2018). In 2014, HWCOM joined the AAMC Core EPA Pilot and endeavored to develop curriculum and assessment based on the EPA framework. Successful implementation of EPAs in the workplace requires institutional engagement, a thoughtful curricular approach, faculty development, and feasible, valid workplace based assessments. Guided by these principles, we describe how we moved from teaching and learning of motivational interviewing (MI) in the classroom to meaningful application and assessment in the workplace using an EPA based framework.

### Motivational Interviewing

Patient-centered behavioral guidance to improve patient self-management, recognized as an evidence-based intervention for a variety of conditions ( Lundahl *et al.*, 2013; O’Halloran *et al.*, 2014; Lindson-Hawley, Thompson and Begh, 2015; Ekong and Kavookjian, 2016; Palacio *et al.*, 2016), involves interpersonal and communication skills such as 1) empathetic understanding of patient views of problems and related biopsychosocial concerns and health behaviors; 2) supporting autonomy; and 3) collaborative medical decision-making ( Abramowitz *et al.*, 2010; Haeseler *et al.*, 2011; William Miller and Rollnick, 2013).

MI “is a patient-centered, goal-oriented style of communication with particular attention to the language of change. It is designed to strengthen personal motivation for and commitment to a specific goal by eliciting and exploring the person’s own reasons for change within an atmosphere of acceptance and compassion” ( William Miller and Rollnick, 2013). A basic theoretical foundation of MI is that “People tend to become more committed to that which they hear themselves defend” ( Hettema, Steele and Miller, 2005). MI involves eliciting and reflecting patient language about change. Evidence shows that MI fidelity impacts patient language and that patient language predicts behavior ( Magill et al., 2018).

Brief MI conversations are valued by practitioners ( Foster et al., 2016; Chisholm et al., 2017; Roman-Rodriguez et al., 2017), fit into everyday practice, and often take no more time than traditional advice giving ( Rubak et al., 2006). Systematic reviews demonstrate positive outcomes for a variety of conditions ( Lundahl et al., 2013; O’Halloran et al., 2014; Lindson-Hawley, Thompson and Begh, 2015; Ekong and Kavookjian, 2016; Palacio et al., 2016). Yet, data on what elements of MI are associated with positive outcomes is limited, and longer MI interventions may not necessarily be more effective than brief ones ( Lindson-Hawley, Thompson and Begh, 2015), or more effective than other forms of counseling. Recent work indicates potential to improve MI efficacy in combination with cognitive behavioral therapy (CBT) ( Riper et al., 2014) or health education ( Gance-Cleveland, 2007). Patients often present with multiple, inter-related behavioral targets or a variety of chronic disease processes ( Butler et al., 2013). Thus, it may be more useful to think of MI as a way of being and communicating with patients about behavior change, flexibly and iteratively, within the context of clinical encounters.

Physicians engage in thousands of encounters throughout their careers, often with multiple interactions per patient annually, presenting a substantial opportunity to promote behavioral health. Nonetheless, physicians often feel unprepared to engage in behavior change conversations, resulting in frustration ( Rushforth et al., 2016). MI offers strategies to approach these conversations in a way that has the potential to facilitate patient and provider satisfaction ( Dunhill, Schmidt and Klein, 2014; Pollak et al., 2016). Furthermore the MI focus on active reflective listening and partnership has significant overlap with strategies for teamwork and leadership ( Frich et al., 2015; McEwan et al., 2017).

### Undergraduate Medical Education MI Curriculum Development

MI is a skill learned through deliberate practice and individualized feedback ( William Miller and Rollnick, 2013). Competence gained during the traditional workshop format tends to diminish quickly without some systematic post-test support or supervision ( W. R. Miller *et al.*, 2004; Martino *et al.*, 2007; William Miller and Rollnick, 2013). While students’ knowledge, confidence, and skills around behavior change counseling have shown improvement through lectures and workshops, on-going reinforcement of skills in authentic clinical settings has been a missing component of MI training programs ( Barwick *et al.*, 2012). Studies of MI training for health care professionals have demonstrated a variable degree of improvements in MI skills, but few studies objectively measure MI skills and none include long term follow-up data ( Barwick *et al.*, 2012; Dunhill, Schmidt and Klein, 2014).

### MI Competency Assessment

The Motivational Interviewing Network of Trainers (MINT), an international organization of trainers in MI founded in 1997, aims to serve as a forum “to promote good practice in the use, research and training of motivational interviewing” ( Motivational Interviewing Network of Trainers, 2018). A number of rating systems have been developed. The Motivational Interviewing Treatment Integrity (MITI) coding instrument is often used to measure MI skills and includes global ratings for “MI Spirit” such as empathy, collaboration, evocation, direction, and autonomy support; and has undergone a number of updates over the years ( Moyers *et al.*, 2005; Moyers *et al.*, 2010; Moyers, Manuel and Ernst, 2014). The MITI also contains technique behavior counts (e.g., a reflection to question ratio; percentage complex [vs. simple] reflections; and an MI non-adherent statement count). The MITI also identifies expert-defined proficiency and competency benchmark scores for both the technique behavior counts and global ratings. The MITI has demonstrated good to excellent reliability and good sensitivity in detecting improvements in skills ( Moyers *et al.*, 2005).

## Methods

### MI Training Methods

Initially lacking a sufficient cadre of trained faculty, we developed faculty in stages, iteratively, alongside training of students. A core group of clinical educators (family medicine, internal medicine, and psychiatry) and a MINT trainer (social work) developed the initial activities and trainer guides. We trained an initial group of clinical educator faculty on MI concepts to serve as small group facilitators. We initially paired those faculty without baseline MI proficiency with volunteer MI-trained social work graduate students to co-facilitate. In subsequent years, these clinical educator faculty led groups themselves and helped train additional faculty. This provided faculty with skills to implement the clinical skills curriculum.

We integrated a primary series of three four-hour workshops into the first-year clinical skills course ( Brogan Hartlieb *et al.*, 2016), supplemented by a single four-hour refresher session one year later. Students were then required to practice MI during household visits beginning at the end of the first year, receiving feedback from faculty based on direct observation. Students also completed an Observed Structured Clinical Examination (OSCE) prior to and after the clerkship year, (i.e., one and two years after initial training).

Students recorded 5 to 10 minute sample role plays prior to and following the first two workshops. Students then each completed an OSCE targeting smoking cessation at one- and two-year follow-up. Although we required students to practice MI during household visits, and trained faculty through workshops, the clinical faculty did not feel effective in providing feedback to students in the clinical environment. In 2016 we undertook a coaching program to enhance faculty skills for providing individualized feedback to students in clinical settings. Participating faculty completed telephone practice and coaching sessions with a MINT trainer. We present findings from both the student MI training and the faculty coaching program.

### Student MI Training Study Methods

Sixty-one out of 80 students from the class of 2015 agreed to participate in a study allowing us to formally examine changes in skills from pre-test to post-test (role plays) and at the two-year OSCE. We collected these competency data from 2012 through 2014. We used audio recording for skills coding. Trained coders applied an adaptation of the MITI (Version 3.1.1) ( Moyers *et al.*, 2010) to measure participant MI skills compared with competency benchmarks, as demonstrated in the pre- and post-test role play and the two-year follow-up OSCE audio recordings.

We tested whether mean MI skills scores would increase at post-test and continue to increase or maintain at the two-year follow-up by measuring changes across assessment periods in MITI global ratings, which is a Likert, 1-5 scale for each empathy, collaboration, supporting autonomy, and evocation. The MITI also includes technique behavior counts (e.g., MI non-adherent statements, total reflection to total question ratio, and complex (versus simple) reflection percentage). We also assessed the percentage of students that met competency benchmarks at pre, post, and two-year follow-up by examining 1) if individual participants scored at least 4 on all global ratings; 2) exceeded a 2:1 or 1:1 total reflection to questions ratio; 3) used at least 50% complex [vs. simple] reflections; and 4) used no MI non-adherent statements.

### Faculty MI Coaching Study Methods

Given limited comfort among clinical faculty in providing feedback during supervision of clinical application of MI skills, we undertook a coaching program to further develop clinical faculty in 2016. Thirteen faculty received between 4-14 (mean = 10) half-hour MI coaching calls over a four-month period. Before and after the coaching, these clinical educator faculty were assessed via video or audio taped clinical interaction with a standardized patient. MINT trainers coded these interactions with the MITI. By 2016, an updated MITI Scale (Version 4.2.1) had been published to account for evolution of MI concepts ( Moyers, Manuel and Ernst, 2014). MITI 4.2.1 contains new “technical” global MI spirit scales of cultivating change talk and softening sustain talk and retains the “relational” global scales for collaboration or partnership, supporting autonomy, and empathy. It retains technique behaviors counts for calculating percentage of complex reflections, and the reflection-to-question ratio, but changed the name of the “proficiency” and “competency” benchmarks to “fair” and “good.”

### Methods for Development of an MI EPA and WBA

Our core clinical educators (for both small groups and household visits) had experience with an adaptation of the MITI for small group activities and OSCE. Nonetheless, faculty were not able to effectively apply the MITI in the household visit setting because the attention required for detailed counting of behaviors was challenging in the context of simultaneously providing clinical oversight. Thus, real-time WBA and feedback using the MITI in the context of a clinical encounter was not feasible to implement.

In 2014, HWCOM joined the AAMC Core EPA Pilot and we have been adapting our curriculum to integrate the EPA framework ( Lomis *et al.*, 2016). It became apparent that an MI interaction is an observable, discrete task that can be entrusted to a learner and thus can be framed as an EPA. To develop a WBA for an EPA based on MI, we followed a process to create a one-page description of the activity modeled on that of the Core EPA Pilot ( Obeso *et al.*, 2017). First, we defined four key functions based on the MITI. We used MITI descriptions to draft developmental schematic towards competency for each function. We used an iterative process of review by MINT trainers, medical educators, and clinical supervisors to develop a one-page schematic that we subsequently adapted and used as a WBA.

## Results

### Student MI Training Study Results

#### Student Global spirit scores

Generalized linear mixed modeling was used to examine the level of participant competency across time for the global scales and behavior counts ratios. We used PROC GLIMIX in SAS 9.4 to model two binary variables: Global competence scale and therapist behavior counts. Missing data due to technical problems with recordings resulted in a sample size of 36 to 33 participants for certain analyses ( Table 1).

**Table 1.** Percentage of Herbert Wertheim College of Medicine Medical Students Meeting MITI 3.1.1 Competency Benchmarks 2012-2014

	Preintervention		Postintervention		Two-Year Follow-up	
	n = 36		n= 33		n = 35	
	%	n	%	n	%	n
All Global Ratings	25	9	24	8	*69	24
No MI Non-Adherent Statements	50	18	67	22	*91	32
Complex to Total Reflection Percentage	33	12	24	8	46	16
Total Reflection to Question Ratio	11	4	24	8	0	0

* *Statistically significant, p <.01*

We found no significant differences on the mean global spirit scores among pre, post and two-year follow-up assessments. However, the percentage of students scoring 4 or 5 on all four global ratings, which was 25% (9/35) at pre-test and 24% (8/33) at post-test, increased significantly to 69% (24/35) at the two-year follow-up.

Twenty five students (69%) crossed the threshold from scores of 3 or below to at least 4 at the two-year follow-up. Eleven students (31%) never met the competency benchmark of 4 or 5 on the global scales; their scores remained the same or declined. Given the small sample size, the scores of these 11 students offset the gains of the other 25 students enough to preclude a statistically significant increase in mean scores overall. The result of modeling the global competence scale shows that the odds of students meeting the global competence threshold significantly increased over time (β = 2.03, p <.01, *Exp*(β)= 7.61). That is, at the two-year follow-up session, the odds of meeting the global competence threshold was 7.61 times greater than at pre or post-test time points.

#### Student Technique behavior count scores

MI training elicited a statistically significant reduction in MI non-adherent statements at two-year follow-up, but not immediately after training. The percentage of students who made no MI non-adherent statements was 50% (18) at pre-test, 67% (22) at post-test, and 91% (32) at two-year follow-up. A significant increase in proportion of reflection statements immediately after training occurred, but was not sustained at two-year follow-up. The study demonstrated that three, four-hour workshops followed by limited opportunity for clinical application and an additional four-hour refresher workshop one year later, can increase UME student MI skills. The study also demonstrated that although most students met the MITI global spirit competency standard and refrained from MI non-adherent statements, they fell well short of the competency standard for using more reflections than questions and using more complex than simple reflections, which are key MI techniques.

### Faculty MI Coaching Study Results

#### Faculty Global relational and technical spirit scores

The small sample size precluded the use of parametric statistics but we present the raw scores. Following the coaching program, faculty demonstrated gains in the relational MI global spirit scores from 54% (7/13) at baseline scoring at least “fair” to 100% (13/13) doing so after four months of coaching ( Table 2). For the technical MI global spirit scores, 92% (12/13) scored at least fair at baseline versus 100% (13/13) doing so after the coaching program.

**Table 2.** Percentage of 13 Herbert Wertheim College of Medicine Faculty Meeting MITI 4.1.1 Competency Benchmarks Before and After Coaching in 2016

	MITI “Fair”				MITI “Good”			
	Baseline		4 month		Baseline		4 Month	
	%	n	%	n	%	n	%	n
Relational	53%	7	100%	13	38%	5	69%	9
Technical	92%	12	100%	13	46%	6	77%	10
% Complex Reflections	53%	7	85%	11	46%	6	62%	8
Reflection: Question Ratio	0%	0	77%	10	0%	0	15%	2

#### Faculty Technique behavioral count scores

Prior to the coaching program, no faculty member study participant reached the beginner-level reflection-to-question ratio of 1:1. After four months of the coaching program, 77% (10/13) of faculty members achieved at least a 1:1ratio, and 15% (2/13) of faculty achieved a 2:1 ratio. The number of faculty members reaching competency levels forcomplex reflections also increased after four months with 7/13 (54%) faculty using 40% or more complex reflections at baseline and 11/13 (85%) faculty after.

Consistent with other studies, coaching increased skills and comfort with MI. The coaching program included key aspects of competency-based skills training (i.e., individualized training and feedback). This style of training required considerably more MINT trainer time than group workshops and clinical faculty found it superior for attaining the skills to coach students in the workplace.

### MI EPA and Workplace-Based Assessment (WBA) Results

**Figure 1.**
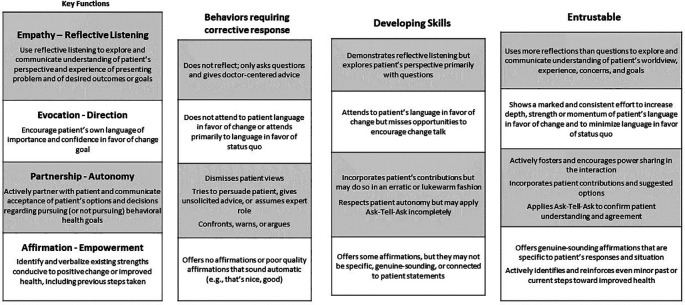
Collaborative Behavioral Guidance Entrustable Professional Activity (Based on Motivational Interviewing)

We developed a one-page schematic ( Figure 1) of an MI EPA by adapting the MITI to the format of the Core EPA Pilot Toolkit.

We subsequently adapted the EPA schematic for use as a WBA. ( Table 3)

**Table 3.** Workplace Based Assessment for Motivational Interviewing Entrustable Professional Activity

**1) Use reflective listening to explore and communicate understanding of patient’s perspective and experience of presentation problem and of desired outcomes and goals**
□ Does not reflect; only asks questions and gives doctor-centered advice
□ Demonstrates reflective listening but explores patient’s perspective primarily with questions
□ Uses more reflections than questions to explore and communicate understanding of patient’s worldview, experience, concerns, and goals
**2) Encourage patient’s own language of importance and confidence in favor of change goal.**
□ Does not attend to patient language in favor of change or attends primarily to language in favor of status quo.
□ Attends to patient’s language in favor of change but misses opportunities to encourage change talk. May struggle to transition to planning phase
□ Shows a marked and consistent effort to increase depth, strength or momentum of patient’s language in favor of change and to minimize language in favor of status quo. Elicits specific, measurable, achievable, realistic, and time limited action plans
**3) Actively partner with patient and communicate acceptance of patient’s options and decisions regarding pursuing (or not pursuing) behavioral health goals.**
□ Dismisses patient views. Tries to persuade patient, gives unsolicited advice, or assumes expert role. Confronts, warns, or argues
□ Incorporates patient’s contributions but may do so in an erratic or lukewarm fashion. Respects patient autonomy but may apply Ask-Tell-Ask incompletely
□ Actively fosters and encourages power sharing in the interaction. Applies Ask-Tell-Ask to confirm patient understanding and agreement. Incorporates patient contributions in the follow up plan.
**4) Identify and verbalize existing strengths conducive to positive change or improved health, including previous steps taken.**
□ Offers no affirmations or poor quality affirmations that sound automatic (e.g., that’s nice, good)
□ Offers some affirmations, but they may not be specific, genuine-sounding, or connected to patient statements
□ Offers genuine-sounding affirmations that are specific to patient’s responses and situation. Actively identifies and reinforces even minor past or current steps toward improved health
**5) How Much Help Did the Learner Require for the Collaborative Behavioral Guidance Activity?**
□ “I did it” - Student required complete guidance or was unprepared; I had to do most of the work myself.
□ “I talked them through it” - Student was able to perform some tasks but required repeated directions.
□ “I directed them from time to time” - Student demonstrated some independence; only required intermittent prompting.
□ “I was available just in case” - Student functioned independently, only needed assistance with nuances or complex situations.
**Feedback** **Strong Points:**
**Attention Suggested:**

The shared mental model of an MI EPA, and related WBA, has helped to focus students and faculty on the observable entrustable behaviors expected in MI conversations. Students are each required to be assessed using the MI WBA via Qualtrics iPad app once each year during household visits. The first year of use yielded 202 assessments and prompts for workplace coaching and feedback, with entrustable range ratings varying across functions: affirmations 90% (182), partnership 83% (167), evocation 77% (156), reflections 66% (133), and supervision 63% (127). These findings corroborate prior observations from faculty that effective use of reflection is one of the most challenging skills to master in MI. And, that the global “supervision level” rating had greater spread than ratings for individual functions of the activity.

## Discussion

Previous MI training studies in medical schools yielded positive but short-term effects ( Barwick *et al.*, 2012; Dunhill, Schmidt and Klein, 2014). The two-year follow-up MI training skills results we report are the longest we have found. Overall, the MI curriculum successfully exposed medical students to patient-centered behavioral guidance and shared decision-making communication skills. More than two thirds of students met competency benchmarks on all global ratings at two-year follow-up and abstained from MI non-adherent statements. These students have demonstrated core skills for promoting engagement and providing self-management support. Still, the student participants did not meet benchmarks for the use of reflections, a key MI technique.

A missing component of our initial training was individualized feedback from fully trained faculty in the field. Although students see patients under faculty supervision, the faculty members’ own MI training was limited. We undertook a second MI training study, this time with faculty, to address this deficit. Findings from this study indicated that most faculty members met the “good” global MI spirit benchmark and the “fair” MI technique benchmark for their use of reflections.

Although the MITI has been used in numerous studies and has demonstrated sound psychometrics ( Moyers *et al.*, 2005), MITI standards are based on expert opinion rather than empirical testing that includes patient behavioral outcomes. The degree to which specific benchmarks defined by the MITI (i.e. 2:1 reflection to question ratio) relate to patient outcomes has not been established. Students and faculty frequently asked about the clinical significance of the expert-defined 2:1 and 1:1 reflection to question ratio benchmarks. Given the challenge that students and faculty had achieving this ratio despite their gains in other MI skills and the lack of research testing this specific question, it is unclear to the extent to which we should strive to meet the 2:1 versus the 1:1 standard. Another issue that arose is that the MITI is not a practical tool for application by a clinical supervisor in the workplace.

We developed the MI EPA based on the MITI with input from four MINT trainers and multiple MD educators. The EPA schematic facilitates a shared mental model for students and faculty of the key functions of MI, offers guidance for authentic workplace skills practice, and appears to offer a more practical WBA than existing frameworks. Initial applications of the MI WBA indicate that it discriminates among skills similar to the MITI.

## Conclusions

Because learning the complex MI skillset requires a repeated practice and feedback, and competence must ultimately be demonstrated in authentic clinical encounters; a thoughtful outcome-based approach, a commitment to faculty development, and assessment tools practical for use in the clinical setting are necessary. We propose an EPA WBA based on MI as a tool for translating MI skill development and assessment from the classroom to the workplace.

Brief, patient-centered behavioral counseling can reduce the cost and negative outcomes of chronic disease. Regardless of specialty, health care is increasingly a team endeavor, impacted by factors outside the control of the physician. Communication, collaboration, and coaching are foundational skills for future physicians. MI is a core EPA for future physicians involving integrated competencies for collaborative care, featuring reflective listening, affirmation, evocation, and collaborative planning. These skills are critical for patient care, and for leading increasingly complicated healthcare organizations, particularly in working with underserved populations.

## Take Home Messages

• An MI interaction is an observable, discrete task that can be framed as an EPA.
• Motivational Interviewing (MI) is a core Entrustable Professional Activity (EPA) for future physicians involving integrated competencies for collaborative care, featuring reflective listening, affirmation, evocation, and collaborative planning.
• Successful implementation of EPAs in the workplace requires institutional engagement, a thoughtful curricular approach, faculty development, and feasible, valid workplace based assessments (WBA).
• We present an EPA one-page schematic and WBA based on established MI competencies.

## Notes On Contributors

**Brett Engle**, PhD, LCSW,is an Assistant Professor in the Department of Humanities, Health, and Society (HHS) at Florida International University (FIU) Herbert Wertheim College of Medicine (HWCOM), Clinical Director at Harbor Village Treatment Center, and a member of the Motivational Interviewing Network of Trainers (MINT).

**Kathryn Brogan-Hartlieb**, PhD, RDis an Assistant Professor in the Department of HHS at HWCOM, a nutrition and communication specialist, and a member of the MINT. Her scholarship focuses on exploring health behavior change with clients, learners and organizations in the fields of nutrition and public health.

**Vivian T. Obeso**, MD,is the Associate Dean for Curriculum and Medical Mducation, and Associate Professor in the Division of Internal Medicine in the Department of HHS at HWCOM. She previously developed the HWCOM clinical skills curriculum and currently has responsibility for the overall medical education program.

**Maryse Pedoussaut**, MD,is Assistant Professor in the Division of Family and Community Medicine in the Department of HHS at HWCOM. She teaches medical students at all levels, with a focus on clinical skills, MI, interprofessional household visits, integrative medicine, and humanism. ORCID: https://orcid.org/0000-0001-5890-7578

**Michelle M. Hospital**, PhD, is Associate Director and Research Associate Professor at the Community-Based Research Institute (CBRI) at FIU. She is an applied life-span developmental psychologist and a licensed mental health counselor with extensive clinical and research experience with adolescent and young-adult, ethnically diverse, inner city populations.

**Carla S. Lupi**, MD, is Associate Dean for Assessment and Evaluation at Kaiser Permanente School of Medicine. She was previously Assistant Dean for Teaching and Learning and Associate Dean for Faculty at HWCOM. Her scholarship covers instruction and assessment in a range of competencies, including communication skills. ORCID: https://orcid.org/0000-0001-5200-6074

**Karin C. Esposito**, MD, PhD,is Executive Associate Dean for Student Affairs and Professor of Psychiatry and Behavioral Health at HWCOM. She was previously Associate Dean for Curriculum and Medical Education and Associate Dean for Academic Affairs at HWCOM.ORCID: https://orcid.org/0000-0002-3988-235X

**David R. Brown**, MD, is Vice Chair, Chief of the Division of Family and Community Medicine, and Associate Professor in the Department of HHS at HWCOM. His research interests include medical education, entrustment, collaboration, population health, and integrating social and behavioral services into healthcare. ORCID: https://orcid.org/0000-0002-5361-6664
