# Supplementary material for: From the Classroom to Entrustment - The Development of Motivational Interviewing Skills as an Entrustable Professional Activity
Source: MedEdPublish (2016). 2019 Jul 17;8:153. [Version 1] doi: 10.15694/mep.2019.000153.1 (PMC10911765; doi:10.15694/mep.2019.000153.1)
Supplement: Peer review history

# Reviewer report: Reviewer response for version 1

**DOI:** 10.21956/mep.19885.r30084

**Published:** 2019-08-20

**Authors:** Yingzi Huang

**Affiliations:** 1 The First Affiliated Hospital, Sun Yat-sen University

This review has been migrated. The reviewer awarded 5 stars out of 5 I enjoyed reading this article very much. It highlights the importance of MI as a crucial technique for patients'behavioral change, with practical consideration of EPA and WBA to integrate MI into medical education for the students. This technique is now more important than ever as the global burden of disease shows the prevalence of chronic diseases, which is probably why it's highly recommended in Australia's red book.MI is worth learning, albeit very difficult and time consuming to grasp. I would suggest the authors to provide more details especially when it comes to 'training the trainers'. For example, whether the coaches/facilitators are properly trained for a sufficient time period and whether they've got qualifications in this area or not. And I wonder if a comparison group is needed, considering that the score's change could be due to other reasons rather than just MI training.

Reviewer Expertise:

NA

No decision status is available

# Reviewer report: Reviewer response for version 1

**DOI:** 10.21956/mep.19885.r30082

**Published:** 2019-08-19

**Authors:** Trevor Gibbs

**Affiliations:** 1 AMEE

This review has been migrated. The reviewer awarded 5 stars out of 5 As someone who is a great fan of motivational interviews, I was pleased to be asked to review this paper- thank you.I have never understood why MI has not taken its rightful place in the undergraduate medical curriculum and I am pleased to see that this paper supports its inclusion.This is a very well researched and structured paper that takes the reader through a very clearly defined process- taking a concept such as MI through an acceptable teaching activity and placing it firmly into a workplace-based activity and EPA. The references alone provide a great foundation to build an interest in MI and I believe this as a paper that should be read by all curriculum planners

Reviewer Expertise:

NA

No decision status is available

# Reviewer report: Reviewer response for version 1

**DOI:** 10.21956/mep.19885.r30083

**Published:** 2019-07-18

**Authors:** Felix Silwimba

**Affiliations:** 1 University of Lusaka

This review has been migrated. The reviewer awarded 5 stars out of 5 this article has provided me with a broader appreciation of the depth of medical education on taken for granted areas of patient communication and behavioral change. Agree this techniques will be useful to those working in under-served populations.

Reviewer Expertise:

NA

No decision status is available
